# Hydrocarbon-Degrading Bacteria *Alcanivorax* and *Marinobacter* Associated With Microalgae *Pavlova lutheri* and *Nannochloropsis oculata*

**DOI:** 10.3389/fmicb.2020.572931

**Published:** 2020-10-28

**Authors:** Tatyana N. Chernikova, Rafael Bargiela, Stepan V. Toshchakov, Vignesh Shivaraman, Evgenii A. Lunev, Michail M. Yakimov, David N. Thomas, Peter N. Golyshin

**Affiliations:** ^1^School of Natural Sciences, Bangor University, Bangor, United Kingdom; ^2^CEB-Centre for Environmental Biotechnology, Bangor University, Bangor, United Kingdom; ^3^National Research Centre “Kurchatov Institute”, Moscow, Russia; ^4^Institute of Living Systems, Immanuel Kant Baltic Federal University, Kaliningrad, Russia; ^5^Institute for Marine Biological Resources and Biotechnology of the National Research Council, IRBIM-CNR, Messina, Italy; ^6^School of Ocean Sciences, Bangor University, Menai Bridge, United Kingdom

**Keywords:** photobioreactor, microalgae-associated bacteria, hydrocarbon-degrading bacteria, 16S rRNA gene amplicon, isolation of oil-degrading bacteria

## Abstract

Marine hydrocarbon-degrading bacteria play an important role in natural petroleum biodegradation processes and were initially associated with man-made oil spills or natural seeps. There is no full clarity though on what, in the absence of petroleum, their natural niches are. Few studies pointed at some marine microalgae that produce oleophilic compounds (alkanes, long-chain fatty acids, and alcohols) as potential natural hosts of these bacteria. We established Dansk crude oil-based enrichment cultures with photobioreactor-grown marine microalgae cultures *Pavlova lutheri* and *Nannochloropsis oculata* and analyzed the microbial succession using cultivation and SSU (16S) rRNA amplicon sequencing. We found that petroleum enforced a strong selection for members of Alpha- and Gamma-proteobacteria in both enrichment cultures with the prevalence of *Alcanivorax* and *Marinobacter* spp., well-known hydrocarbonoclastic bacteria. In total, 48 non-redundant bacterial strains were isolated and identified to represent genera *Alcanivorax*, *Marinobacter*, *Thalassospira*, *Hyphomonas*, *Halomonas*, *Marinovum*, *Roseovarius*, and *Oleibacter*, which were abundant in sequencing reads in both crude oil enrichments. Our assessment of public databases demonstrated some overlaps of geographical sites of isolation of *Nannochloropsis* and *Pavlova* with places of molecular detection and isolation of *Alcanivorax* and *Marinobacter* spp. Our study suggests that these globally important hydrocarbon-degrading bacteria are associated with *P. lutheri* and *N. oculata*.

## Introduction

Marine microalgae are an important nutritional source and microenvironment for the growth of diverse marine bacteria (Reynolds, 2006; Amin et al., 2015; Krohn-Molt et al., 2017). It has been shown that as primary producers, microalgae allow bacteria to have access to substrates that are produced by algae such as oxygen, dissolved organic matter, and exopolymeric substances in exchange for macro- and micro-nutrients, such as vitamin B_12_, carbon dioxide, and iron (Fe-siderophore complexes), thereby supporting algal growth (Hulatt and Thomas, 2010; Cooper and Smith, 2015). In nature, relationships between microalgae and bacteria are not limited to the exchange of metabolites but may include ecological interactions ranging from parasitism to mutualism resulting in lateral gene transfer (Kouzuma and Watanabe, 2015; Ramanan et al., 2015). These interactions are well-studied and recognized as a vital part of algae-bacterial interactions that not only influences the growth of bacteria associated with microalgae but also determines the overall success of microalgae themselves (Fuentes et al., 2016; Seymour et al., 2017).

Studies of the diversity of microalgae-associated bacterial communities have demonstrated that the composition of microbial communities varies between different phytoplankton hosts (Teeling et al., 2012; Goecke et al., 2013; Buchan et al., 2014; Krohn-Molt et al., 2017; Seymour et al., 2017). Furthermore, a few studies have also reported that some microalgae co-exist with hydrocarbon-degrading bacteria [or hydrocarbonoclastic (HCB)] bacteria, such as *Alcanivorax* spp. and *Marinobacter* spp. (Green et al., 2004, 2006, 2015; Yakimov et al., 2007; Amin et al., 2009; McGenity et al., 2012). Strains with close phylogenetic affiliation to either *Alcanivorax borkumensis*, the most ubiquitous HCB bacterium, or to *Marinobacter hydrocarbonoclasticus* (genus *Marinobacter*), have been isolated from dinoflagellate *Gymnodinium catenatum* cultures originating from different geographical locations (Green et al., 2004, 2006). The high percentage of members of the genus *Marinobacter* was detected in cultures of dinoflagellate and coccolithophorid (Amin et al., 2009; Green et al., 2015). Those studies have led to a discovery of several new genera and species of polyaromatic-degrading bacteria, e.g., *Porticoccus hydrocarbonoclasticus*, *Polycyclovorans algicola*, *Algiphilus aromaticivorans*, and *Arenibacter algicola* (Gutierrez et al., 2012a,b, 2013, 2014). The isolation, description, and in some cases, whole-genome sequencing of these microorganisms have provided a basis for the understanding of the networks of polyaromatic hydrocarbon (PAH)-degrading microalgae-associated bacteria (Gutierrez et al., 2015, 2016). The mechanisms that could explain the association of HCB degraders with phytoplankton are not well understood, although some explanations have been considered in several works (Mallet and Sardou, 1964; Andelman and Suess, 1970; Gunnison and Alexander, 1975; Marlowe et al., 1984; Shaw et al., 2003; Exton et al., 2012). These studies demonstrated that some microalgae synthesized long-chain alkenones and isoprene-volatile hydrocarbon and accumulated and/or absorbed PAHs. However, there is no full clarity about the association of hydrocarbon-degraders with phytoplankton in marine environments as possible natural niches. Recently, Thompson et al. (2017) showed natural phytoplankton-associated bacterial communities exposed to oil changed in community structure toward one with increased numbers of species responsible for the degradation of crude oil. Nevertheless, our knowledge of the range of microalgae that can host hydrocarbon-degraders, the composition of microalgae-associated microbial consortia, and responses of these consortia to the amendment of petroleum is still incomplete.

Studies on microalgae-associated hydrocarbon-degrading microbial communities have only been conducted so far with a handful of microalgae species (Mishamandani et al., 2016; Thompson et al., 2017), and in general microalgal species have not been assessed for their ability to host hydrocarbon-degraders. Many photobioreactor (PBR)-grown microalgal species are a good source of heterotrophic microbial communities (Watanabe et al., 2005; Otsuka et al., 2008; Ueda et al., 2009; Lakaniemi et al., 2012a,b,c; Lee et al., 2013) and potentially, hydrocarbon degraders (Hammed et al., 2016). Studies on phylogenetic characterization of microalgae-associated bacterial communities in PBRs undertaken by Lakaniemi et al. (2012a,b,c) have established microbial consortia predominantly populated by Proteobacteria, in particular, Alphaproteobacteria in cultures of *Chlorella vulgaris* and Alpha- and Gamma-proteobacteria in cultures of *Dunaliella tertiolecta*. Using sequence-based metagenome and functional analysis, Krohn-Molt et al. (2013) found that a bacterial biofilm associated with *C. vulgaris* and *Scenedesmus obliquus* in an outdoor PBR tended to be restricted to 30 bacterial species that were affiliated with Alpha- and Beta-proteobacteria and Bacteroidetes. Recently, some efforts have been undertaken to analyze a microbiome of a microalgae mass culture in secondary-treated wastewater in prototype OMEGA PBRs (Carney et al., 2014).

Considering the huge interest in microorganisms associated with microalgae, this work was conducted to gain first insights into the biodiversity of potential hydrocarbon-degrading bacteria associated with microalgae cultures of the *Pavlova lutheri* (class Pavlovophyceae) and *Nannochloropsis oculata* (class Eustigmatophyceae). The physiology and applications of these microalgae are well-established. For example, it was reported on the ability of *N. oculata* to grow at different salinities, temperatures, and pH conditions (Spolare et al., 2006; Gu et al., 2012). In addition, it produces lipids with high levels of eicosapentaenoic acid (EPA) and is a promising candidate for biotechnological processes (carotenoids and biofuel production; Volkman et al., 1989; Sudjito et al., 2014; Neto et al., 2018). Moreover, the work of Arriada and Abreu (2014) demonstrated the bioremediation potential of *N. oculata* by growing this strain in produced water (PW) generated from oil production. The high growth rate and high concentration of the essential polyunsaturated fatty acids, EPAs, and docosahexaenoic acids (DHAs), were also demonstrated for *P. lutheri* that make it of great biotechnological potential for biodiesel production (Green, 1975; Volkman et al., 1991; Meireles et al., 2003; Amaro et al., 2011; Shah et al., 2014; Alvarez et al., 2017). However, these microalgae species have not been yet examined in detail with respect to the bacterial communities that could co-exist with them. We utilized Illumina MiSeq amplicon sequencing of 16S rRNA gene amplicons (V4 region) and Oxford Nanopore sequencing of full-length SSU rRNA gene amplicons to characterize bacterial community composition in response to the addition of Dansk crude oil. Finally, we isolated and identified a few dozen bacterial hydrocarbon-degrading strains from *P. lutheri* and *N. oculata* cultures that generally matched the data from culture-independent analysis.

## Materials and Methods

### Microalgae

Microalgal species, *P. lutheri* (#931/1, CCAP, Scotland) and *N. oculata* (#352-567-0226, ZM System, Winchester), were cultured in PBRs for 4 weeks. The growth conditions were: working volume 30 L, temperature 22 ± 2°C, and continuous illumination using cool daylight lamps (Osram L 36 W/865 Lumilux, Germany). The cultures were aerated by bubbling air from the bottom of PBRs. Filtered seawater (0.22 μm Millipore filters) supplemented with nutrient and vitamin solutions from Walne’s medium for algal cultures were used as the growth media (Walne, 1970).

### Experimental Set-Up

Samples of the two microalgal cultures (100 ml) were taken in sterile flasks, in duplicates. These original microalgal cultures of *N. oculata* and *P. lutheri* used as inocula to establish enrichment cultures with crude oil were designated as variants “N” and “P,” respectively. Marine broth (ZM/10) supplemented with 1 ml L^−1^ trace elements solution SL-10 (Widdel et al., 1983) and 10 ml L^−1^ Kao and Michayluk vitamins solution (100x; Sigma-Aldrich) and Dansk crude oil (0.1%) were used to set-up enrichment cultures. The composition of marine broth ZM/10 was as follows: 75% autoclaved seawater, peptone (0.05%), and yeast extract (0.01%). Enrichment for each variant was done in duplicate in 250 ml Erlenmeyer flasks with a total volume of 50 ml, i.e., 48 ml of medium and 2 ml of microalgae culture as an inoculum. Control variants were done using the same medium without the addition of crude oil. Variants of oil-amended enrichments with *N. oculata* hereafter are designated as “NO,” and those derived from *P. lutheri* are termed “PO.” Control variants without the addition of crude oil were designated “ECP” for *P. lutheri*, and “ECN” for *N. oculata*. Flasks were incubated with shaking (100 rpm) on the orbital shaker for 3 weeks in an incubator at 20°C in the dark to prevent the growth of microalgae.

### DNA Isolation and PCR Amplification of 16S rRNA Genes

For DNA extraction of the starting inocula, 10 ml samples from the original microalgal cultures of *P. lutheri* and *N. oculata* were initially withdrawn from PBRs. The biomass was concentrated by centrifugation and stored at −20°C until DNA processing. Samples from variants with crude oil and control variants were collected after 3 weeks of incubation. DNA extraction was done using PowerSoil DNA isolation kit (MO BIO) according to manufacturer’s instructions. Duplicates of DNA samples were pooled to prepare only one DNA sample per each variant. An aliquot of each DNA samples was checked for quality and quantity by gel electrophoresis on a 0.8% agarose gel and by measuring the concentration of DNA by Qubit® dsDNA HS kit (Life Technology) with Qubit® Fluorometer. These DNA samples were used as a template for barcoded-amplicon sequencing.

Extracted DNA samples were used to amplify the V4 region of 16S rRNA gene using fusion dual-barcoded primers (Fadrosh et al., 2014) with slight modifications: the rRNA-annealing part of the forward primer corresponded to F515 (5'-GTGCCAGCMGCCGCGGTAA-3'; Turner et al., 1999) and the reverse primer corresponded to R806 (5'-GGACTACHVGGGTWTCTAAT-3'; Caporaso et al., 2011). Reactions were performed in triplicate in a final volume of 25 μl. PCR conditions were as follows: 94°C for 45 s; 50°C for 1 min; and 72°C for 30 s for a total of 30 cycles, followed by a final incubation at 72°C for 5 min. The obtained amplicons were pooled and purified using a QIAEX II DNA Extraction kit (Qiagen). The prepared libraries were sequenced with MiSeq™ of Illumina Inc. (San Diego, CA, United States) using paired-end 250-bp reads.

Furthermore, to amplify and sequence the full-length 16S rRNA gene, we used a 16S Barcoding kit (SQK-RAB204) according to the protocol by Oxford Nanopore Technologies (ONTs). Amplification of PCR products was done using the 27F forward primer (5' AGAGTTTGATCMTGGCTCAG-3') and 1492R reverse primer (5'-TACGGYTACCTTGTTACGACTT-3') with barcodes supplied in the kit. The obtained PCR products were purified using Agencourt AMPure XP beads (Beckman Coulter) according to the Nanopore’s protocol. The purified and pooled in equimolar proportion DNA was subjected to library construction by attachment of rapid 1D sequencing adapters provided in 16S Barcoding kit (Oxford Nanopore). After the priming the flow cell (FLO-MIN106 R9 Version), the prepared library was loaded into MinION^™^ Flow Cells and sequenced during 24 h on MinION^™^ using MinKNOW^™^ workflow.

### Bioinformatic Analysis and Assessment of Phylogenetic Diversity

All reads generated by Illumina MiSeq® were subjected to stringent quality filtering, and parts of reads corresponding to 16S rRNA primers were removed using CLC Genomic Workbench 10.0 (Qiagen, Germany). All reads that did not include 16S rRNA primer sequence were excluded from the further analyses. After adapter removal, paired reads were merged using SeqPrep tool.^1^ Demultiplexing, open-reference operational taxonomic unit (OTU) picking with USEARCH algorithm (Edgar 2010) against Silva128 database (Yilmaz et al., 2014), and diversity analysis were performed with the corresponding script of QIIME package (v1.9.1; Caporaso et al., 2010).

Reads generated by MinION Nanopore were filtered using *filtlong* (v0.2.0, https://github.com/rrwick/Filtlong) using parameters – keep 90%, – min length 1,000 and min mean *q* 10, in order to keep the longest and best quality reads. Filtered reads were then corrected using canu (v2.0, https://github.com/marbl/canu, Koren et al., 2017). The final files with the reads were aligned against the SILVA 16S rRNA (Yilmaz et al., 2014) database using LAST (v1080, http://last.cbrc.jp/). Only those reads that showed a percentage of identity >90% and a minimum alignment length of 250 bases were considered for further analysis. Based on the results from LAST, taxonomic affiliations were assigned to each of the reads according to their best match against the SILVA database. The number of OTUs was obtained by regarding the unique number of affiliations among all reads, discarding those with less than three reads per OTU. The reads affiliated to Chloroplasts were removed from the analysis.

The OTU tables obtained from both sequencing methods were processed to assess the relative abundance of taxonomic groups at the different taxonomic levels. Common and unique affiliations between Illumina and Nanopore at genus level were specifically studied for samples NO and PO.

All statistics and visualization of the sequencing were performed using scripts developed under R software (v3.6.3, http://www.R-project.org/) using the core packages. The predictions for rarefaction curves were developed using the R package *drc* (v3.0.1) following a Yield-Loss curve model (Ritz et al., 2015). Illumina data were also analyzed by fitting a generalization linear model (GLM) for multivariate data using package *mvabund* (Wang et al., 2012). The relative abundance of microbial OTUs (Illumina data) was exposed to non-metric multidimensional scaling (NMDS) using R software package *vegan* (Oksanen et al., 2019). A Bray-Curtis distance matrix was used as a dissimilatory index. For the development of the sequences’ locations map, a search of 16S rRNA gene sequences (in case of *Alcanivorax* and *Marinobacter*) and 18S rRNA gene sequences (for *Nannochloropsis* and *Pavlova*) were made in GenBank specifying the different genera on the term *organism*. Each search was performed by combining the Entrez Programming Utilities (E-utilities) URL sintax and Perl scripts to fetch and organized all the information (Wall, 2010). For those cases where the retrieved sequences did not show the location coordinates on the GenBank file, the manual check of the articles where they were published (when it was possible) was done. Once all coordinates were gathered, the map was developed using *OpenStreetMap* (v0.3.4; Fellows, 2019) and *sp* (v1.4.1; Pebesma and Bivand, 2005) packages.

### Isolation and Characterization of Hydrocarbon-Degrading Bacterial Strains Associated With *P. lutheri* and *N. oculata*

The mineral medium ONR7a (Dyksterhouse et al., 1995) was used to isolate hydrocarbon-degrading bacterial strains. After 3 weeks of incubation of the enrichments, 100 μl aliquots from serial dilutions (10^−2^–10^−5^) of cultures were plated on solid ONR7a mineral medium. Equimolar mixture of dodecane, tetradecane, and hexadecane alkanes fed through vapor, served as a source of carbon and energy. Plates were incubated at 20°C for 2 weeks. Individual colonies were transferred onto fresh agar plates with the same medium several times in order to obtain pure cultures. Finally, all obtained isolates were checked for growth on ONR7a mineral medium (no alkanes added) to remove potential oligotrophs and agar-utilizing bacteria. The 16S rRNA gene of isolates obtained in pure culture was amplified using universal primers 27F and 1492R (Lane, 1991). The reaction conditions were as follows: 5 min at 94°C, followed by 29 cycles at 94°C for 1 min, 50°C for 1 min, and 72°C for 2 min, and a final extension at 72°C for 10 min. The partial sequencing of the obtained PCR products was done at Macrogen Ltd. (South Korea). The partial 16S rRNA gene sequences obtained from isolates were compared with the 16S subset of nucleotide sequences (refseq_rna) in the NCBI database using BLASTn algorithm (Altschul et al., 1990) to find the closest phylogenetic relatives. Chimera formation was checked using the DECIPHER web tool (http://decipher.cee.wisc.edu/FindChimeras.html; Wright et al., 2012). The alignment was calculated using MAFFT (Katoh and Standley, 2013). The neighbor-joining consensus tree was constructed using Tree builder of Geneious (v9.1.3, https://www.genious.com) using genetic distance model Jukes-Cantor with 1,000 replicates for bootstrap analysis.

### Sequence Accession Numbers

The sequences of amplicons from Illumina MiSeq were deposited in NCBI GenBank under accession numbers of SRR10569254-SRR10569259. The 16S rRNA gene amplicon sequences (MinION outputs) have been deposited in NCBI GenBank under accession numbers from SRR10569250 to SRR10569253. The partial sequences of 16S rRNA gene of obtained isolates were deposited in NCBI under accession numbers of MN794514- MN794561.

## Results

### SSU rRNA Gene Amplicon Sequencing Analysis (V4 Region)

**Figure 1:**
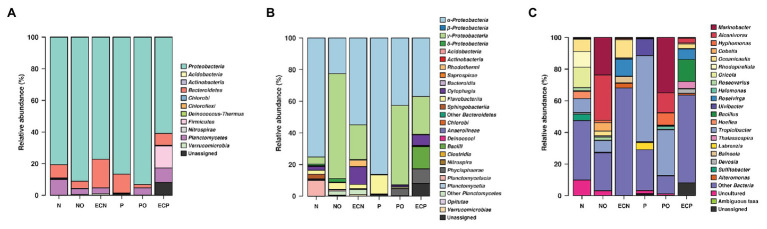
V4 amplicon sequencing data-based community composition at the phylum- **(A)**, class- **(B)**, and genus-levels **(C)** of bacterial communities in samples (% of reads). N, the original culture of *Nannochloropsis oculata*; NO, enrichment of *N. oculata* culture with crude oil; ECN, control of *N. oculata* culture without crude oil; P, the original culture of *Pavlova lutheri*; PO, enrichment of *P. lutheri* culture with crude oil; and ECP, control of. *P. lutheri* culture without crude oil. For data production, biological duplicates of all variants were analyzed.

We detected the following bacterial phyla across all samples: Proteobacteria, Planctomycetes, Bacteroidetes, Verrucomicrobia, Firmicutes, Deinococcus-Thermus, and Actinobacteria (Figure 1A, Supplementary Table S1). Analysis of initial bacterial communities showed that Proteobacteria were the most abundant phylum in both microalgae cultures followed by the Bacteroidetes. Within the Proteobacteria, most amplicon reads originated from organisms from the class Alphaproteobacteria. Planctomycetes were present in the *N. oculata* culture only. Microalgae *N. oculata* (N) hosted the genera *Marinobacter*, *Alcanivorax*, *Oceanicaulis*, *Rhodopirellula*, *Oricola*, *Roseovarius*, *Halomonas*, *Hoefliea*, *Tropicibacter*, *Devosia*, and *Sulfitobacter* while the microbial communities of *P. lutheri* (P) were represented by the genera *Marinobacter*, *Roseovarius*, *Ulvibacter*, *Tropicibacter*, *Thalassospira*, and *Labrenzia* (Supplementary Table S3). The members of hydrocarbonoclastic genus *Marinobacter* were detected in both microalgae species (N and P), whereas the genus *Alcanivorax* was only observed in *N. oculata* (N). Moreover, they represented less than 0.5% of the total number of sequencing reads.

After 3 weeks of incubation, analysis of oil-spiked samples NO and PO demonstrated that the members of the Gammaproteobacteria increased sharply while Alphaproteobacteria decreased in both samples in comparison to the original (P and N) and the control (ECP and ECN) cultures (Figure 1B, Supplementary Table S2). Gammaproteobacteria were almost equally represented between both species, while the Alphaproteobacteria were more abundant in PO enrichment of *P. lutheri*. Within the Gammaproteobacteria, members of the *Alcanivorax* and *Marinobacter* dominated both in enrichments with crude oil and were the two most abundant genera (Figure 1C, Supplementary Table S3). Among other genera, *Halomonas* was enriched only in the PO samples. Moreover, within this class, the minority of sequences in the NO sample were affiliated to the genus *Polycyclovorans* which was not detected in the initial culture of *N. oculata*. The members of the Alphaproteobacteria that were enriched both in NO and PO samples comprised the genera *Hyphomonas*, *Roseovarius*, and *Tropicibacter*. The less abundant genera in the NO samples included *Paracoccus*, *Litorimonas*, and *Altererythrobacter*. The representatives of the Flavobacteriia (phylum Bacteroidetes) were found at low levels in both enrichments with oil and enriched genera included *Owenweeksia*, *Muricauda*, *Arenibacter*, and *Marivirga*. The proportion of members of the phylum Planctomycetes decreased in both PO and NO samples. The sequencing reads related to the classes Planctomycetacia and Phycisphaera (phylum Planctomycetes) were detected only in the NO sample and were affiliated to the genera *Rhodopirellula* and *Algisphaera* (Supplementary Table S3).

### Full-Length SSU rRNA Gene Amplicon Sequence Analysis

**Figure 2:**
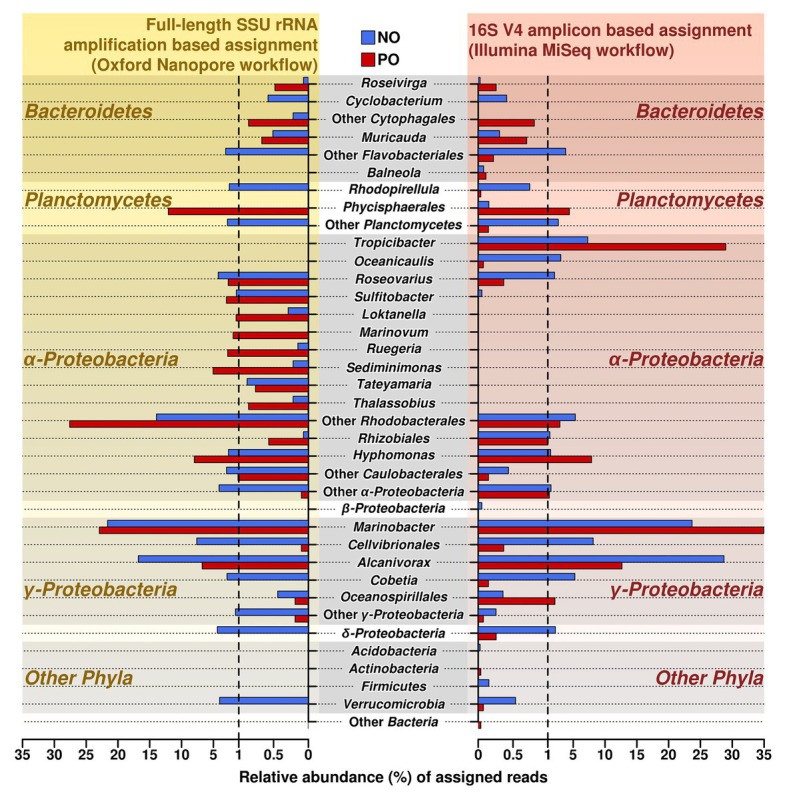
Relative abundance and distribution of reads assigned at the genus level in samples NO and PO, after analysis of data produced by MiSeq Illumina and Oxford Nanopore workflows. NO, enrichment of *N. oculata* with crude oil; and PO, enrichment of *P. lutheri* with crude oil.

To verify and further investigate the sequences at the genus level that we recovered with Illumina short reads 16S amplicons, the full-length 16S rRNA genes Nanopore-based libraries were generated from the same samples. Taking into account the sequencing error rate of long-read Oxford Nanopore Technologies’ MinION platform (Rang et al., 2018), the following processing strategies were applied to long reads from ONT, such as filtering using *fitlong* and error-correction by *canu* to improve read quality for ONT sequencing data. The comparison of both Illumina and Nanopore sequencing data of the oil-enriched microbial communities at the genus level revealed that the majority of 16S barcoding reads obtained by Illumina were detected in the Nanopore sequencing data, but that the proportional distribution of the recovered sequences varied (Figure 2, Supplementary Table S4). Moreover, the results obtained from Nanopore barcoding analyses enabled us to determine more bacterial genera in both enrichments with crude oil.

The number of sequencing reads affiliated to genera *Alcanivorax* and *Marinobacter* (class Gammaproteobacteria) was the highest in both variants, NO and PO, regardless of the amplification and sequencing workflow. Both analyses demonstrated that the number of the sequences belonging to the genus *Alcanivorax* was greater in sample NO while the PO sample contained the larger fraction of sequences belonging to *Marinobacter* spp.

As in the case of MiSeq Illumina, Oxford Nanopore sequencing data also showed that the enrichments NO and PO contained representatives of similar bacterial genera; however, their proportions were different in two microalgae species (Supplementary Table S4). For example, the sequences related to genus *Hyphomonas* were more abundant in the PO than in NO. In contrast to Illumina data, the *Roseovarius* and *Cobetia* showed a higher amount of sequences detected in the samples NO.

Similar microbial communities’ profiles of the enriched genera from class Flavobacteriia phylum Bacteroidetes were detected by Nanopore workflow. Genera *Roseivirga* and *Muricauda* were found both in NO and PO enrichments while genus *Cyclobacterium* was detected only in NO enrichment.

Other genera, though less abundant, identified by both methods in the samples NO and PO are shown in Supplementary Table S4 in the Supplementary Material.

### Microbial Diversity in Microalgae-Associated Bacterial Communities

Bacterial diversity was estimated in all samples using the Shannon index for the partial 16S rRNA amplicons (Illumina and Nanopore experimental data). Supplementary Figure S1 showed the rarefaction curves of sample-based numbers of OTUs together with the curves of prediction-based numbers of OTUs. Even though the initial microalgal culture of *P. lutheri* (P) had the lowest value for bacteria, followed by *N. oculata* (N; Shannon indices 0.58 and 1.26, respectively) in experimental samples using Illumina data, the extrapolation curves based on predicted values for the same samples were higher (Supplementary Figure S1A). A similar pattern was obtained using Nanopore data (Supplementary Figure S1B), where the Shannon indices were 1.97 and 1.35 for initial microalgal cultures of *P. lutheri* (P) and *N. oculata* (N), respectively. In enrichments, the diversity value obtained on Illumina data in sample NO (Shannon index of 2.4) was higher than in PO (Shannon index of 1.94), and the same pattern was identified using an extrapolation curves assessment method. Analysis of Nanopore data showed that Shannon indices for both NO and PO were similar (3.72 and 3.45, respectively). In the case of control samples, ECP and ECN samples Shannon indices were 2.74 and 2.69, respectively.

**Figure 3:**
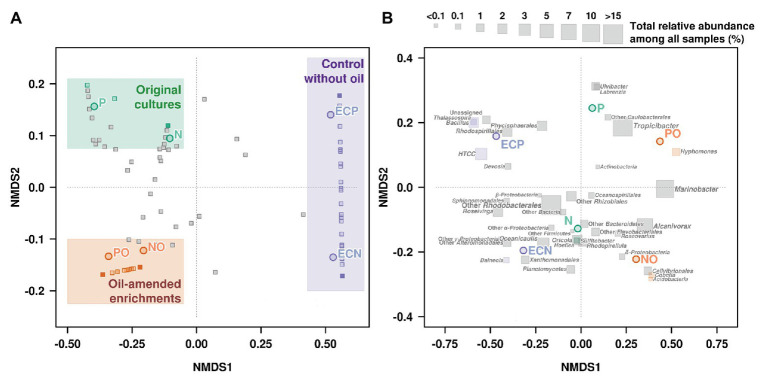
Non-metric multidimensional scaling (NMDS) plot illustrating the relationship **(A)** and distribution **(B)** of OTUs (Illumina data) between microbial communities’ composition in experimental samples. N, the original culture of *N. oculata*; NO, enrichment of *N. oculata* culture with crude oil; ECN, control of *N. oculata* culture without crude oil; P, the original culture of *P. lutheri*; PO, enrichment of *P. lutheri* culture with crude oil; ECP, control of. *P. lutheri* culture without crude oil.

Non-metric multidimensional scaling was used to visualize differences in microbial communities associated with microalgal cultures in response to the oil amendment based on the Bray-Curtis distance matrix (Figure 3, Supplementary Table S6). The obtained patterns for the samples NO and PO were clustered together and were distinct from the control samples and the original microbial communities (Figure 3A). Moreover, NMDS analysis was also performed based on OTUs according to their taxonomical assignment and abundance (Figure 3B). The relative abundance of *Marinobacter* and *Alcanivorax* OTUs on the NMDS plot demonstrated a strong correlation with oil amended enrichments NO and PO. The statistical analyses using the GLM and Likelihood ratio test (LRT) applied to the OTUs represented on the NMDS plot showed differences among microbial communities in the original samples, oil amended samples, and controls (GLM, *p* = 0.004, LRT = 203.9).

### Diversity of Microalgae-Associated Hydrocarbon-Degrading Bacterial Isolates

**Figure 4:**
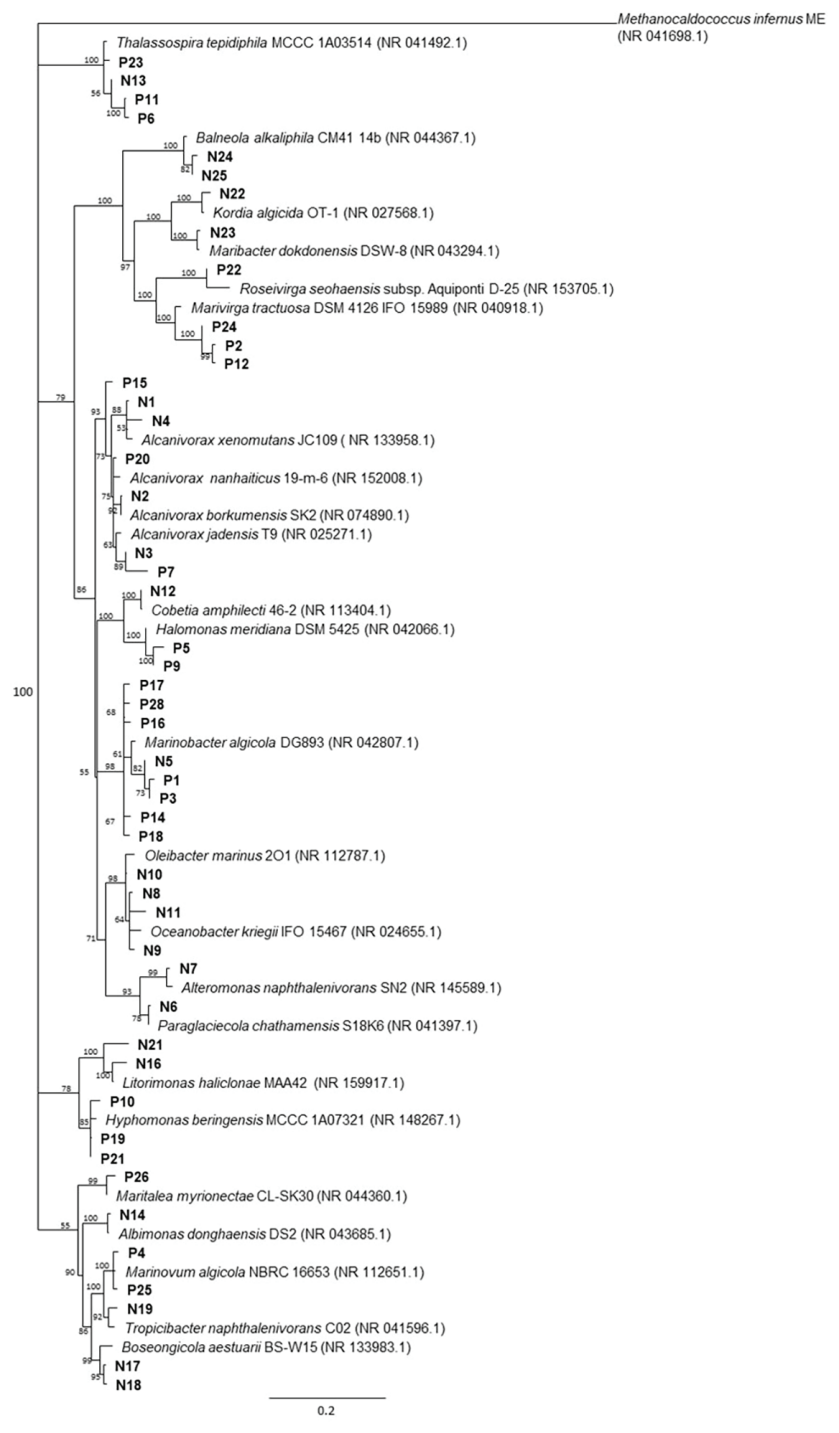
The neighbor-joining consensus phylogenetic tree based on partial 16S rRNA gene sequences of bacterial isolates obtained from oil enrichments. Bootstrap values are 1,000 replicates. References from the 16S rRNA NCBI database; (P) isolates from oil enrichment of *P. lutheri*; (N) isolates from oil enrichment of *N. oculata*. *Methanocaldococcus infernus* ME (NR 041698.1) was used as the outgroup.

Overall, 48 non-redundant strains in total were isolated from enrichment cultures of *P. lutheri* and *N. oculata* enriched with crude oil (Figure 4, Supplementary Table S7). Sequencing of 16S rRNA genes of isolates associated with both microalgal species was assigned to three phyla: Proteobacteria, Bacteroidetes, and Balneolaeota. Within Gammaproteobacteria all isolates belonging to the genus *Marinobacter* in both microalgae cultures showed up to 99.8% 16S rRNA sequence identity with *M. algicola* strain previously isolated from laboratory cultures of dinoflagellates *G. catenatum* and *A. tamarense* (Green et al., 2006). The closest 16S rRNA gene sequences for *Alcanivorax*-related isolates were mostly represented by *A. jadensis* and *A. nanhaiticus* in case of *P. lutheri* and *N. oculata* and *A. xenomutans* and *A. borkumensis* in case of *N. oculata*. Members of the genus *Alcanivorax* were shown to found in phytoplankton-associated HCB communities from the west coast of Scotland and marine diatom *Skeletonema costatum*, and isolated from *E. huxley* and *C. pelagicus f. braarudii* (Green et al., 2004, 2015; Mishamandani et al., 2016; Thompson et al., 2017). Besides isolates of the genera *Alcanivorax* and *Marinobacter*, other Gammaproteobacteria obtained from *N. oculata* were represented by isolates of genera: *Oleibacter*, *Oceanobacter*, *Cobetia*, and *Alteromonas*. The isolates obtained from *P. lutheri* and belonging to the *Halomonas* genus have 98–99% 16S rRNA gene sequence identity to *H. meridiana*. The isolates obtained from *P. lutheri* within the class Alphaproteobacteria were affiliated to genera *Thalassospira*, *Hyphomonas*, *Marinovum*, and *Maritalea*. Closest 16S rRNA gene sequences from the genus *Thalassospira* were related to *T. tepidihila*, a polycyclic hydrocarbon-degrading bacterium isolated from seawater (Kodima et al., 2008). The isolates related to the genus *Hyphomonas* shared a 98% sequence identity with *H. beringensis* isolated from the surface seawater of the Bering sea (Li et al., 2014). The sequences of isolates obtained from *N. oculata* within Alphaproteobacteria were identified as genera *Thalassospira*, *Albimonas*, *Litorimonas*, *Boseongicola*, and *Tropicibacter*. The isolation of members of genera *Thalassospira* and *Hyphomonas* were also reported from *G. catenatum*, *E. huxley*, and *C. pelagicus f. braarudii* (Green et al., 2004, 2015). The other group of isolates was affiliated with the phylum Bacteroidetes class Cytophagia. The isolates within this class had an identity of 94–96% with the strain *Marivirga tractuosa* (genus *Marivirga*), and one isolate had 100% identity with the strain *Rosevirga seohaenaensis* (genus *Roseivirga*; Supplementary Table S7). Sequences of isolates obtained from *P. lutheri* and affiliated with the phylum Bacteroidetes were closely related to the genus *Maribacter* (99% identity with *M. dokdonensis*) and *Kordia* (98% identity with *K. algicida*). Moreover, in our experiment, within the phylum Balneolaeota the sequences of isolates associated with *N. oculata* were identified as members of genus *Balneola* with 97–98% identity with *B. alkaliphilia*.

### Geographic Distribution of Hydrocarbonoclastic Bacteria and Microalgal Hosts

**Figure 5:**
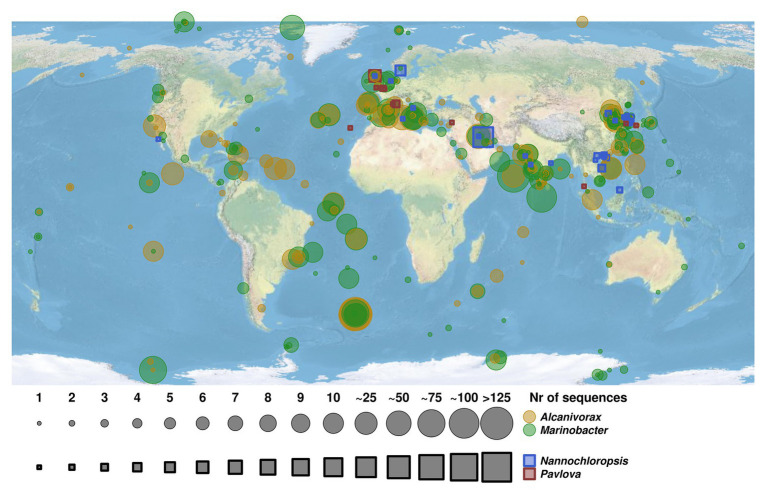
Map of geographic distribution of representatives of hydrocarbon-degrading bacterial genera *Alcanivorax* and *Marinobacter* and their co-occurrence with microalgal species of genera *Pavlova* and *Nannochloropsis*. The size of circles and squares corresponds to the number of retrieved sequences of bacterial strains and microalgae species. The *Alcanivorax*-derived and *Marinobacter*-derived sequences are indicated in yellow and green circles, respectively. The sequences retrieved from microalgae species of genera *Nannochloropsis* and *Pavlova* are indicated in blue and red squares, respectively.

Analysis of the geographic distribution of microalgal species *P. lutheri* and *N. oculata* and hydrocarbonoclastic bacterial strains of the genera *Alcanivorax* and *Marinobacter* is shown in Figure 5. A total of 3,506 sequences for these two oil-degrading bacterial genera and 88 sequences for microalgae were identified in the GenBank (Supplementary Table S8). In comparison to *Alcanivorax* and *Marinobacter*, there is a relatively limited data regarding the isolation, or *in situ* detection of microalgae, nevertheless, *Alcanivorax* and *Marinobacter* co-occurred and/or matched microalgae *P. lutheri* and *N. oculata* isolation sites in most cases, as shown in Figure 5. The experimental evidence to confirm the co-occurrence of these bacteria and microalgal hosts *in situ* at a large scale is therefore yet to be established.

## Discussion

Our study was undertaken with microalgal cultures *P. lutheri* and *N. oculata* to assess their ability to host hydrocarbon-degrading bacteria. Microbial communities co-occurring with *P. lutheri* and *N. oculata* in photobioreactors were spiked with crude oil and subjected to 16S rRNA gene amplicon sequencing analysis. The phylum Proteobacteria was the most prominent in all samples. Gammaproteobacteria was the most abundant class in experiments with crude oil amendment. The majority of bacterial taxa within this class are known hydrocarbon degraders, in particular, the genera *Alcanivorax* and *Marinobacter*. Both represented only 0.5% (or less) of total amplicon reads in the original microalgal cultures *N. oculata* and *P. lutheri*; however, they increased in their relative abundances and became the predominant genera in crude oil enrichments derived from both microalgae (Supplementary Table S3). These results concur with previous studies that demonstrated the significant shift in bacterial community composition in seawater toward *Alcanivorax* and *Marinobacter* spp. after addition of crude oil (Harayama et al., 1999; Kasai et al., 2002; Head et al., 2006; Cappello et al., 2007; McKew et al., 2007; Yakimov et al., 2007); however, those studies assessed changes in microbial composition in seawater or seawater and oxic sediments.

The most abundant sequences within the genus *Marinobacter* obtained in enrichments with crude oil were closely related to the species *Marinobacter hydrocarbonoclasticus* VT8 (100% sequence identity), *Marinobacter hydrocarbonoclasticus* SP17 (100%), and *Marinobacter algicola* DG893 (100%; Supplementary Table S5). *M. hydrocarbonoclasticus* VT8 and *M. hydrocarbonoclasticus* SP17 isolated from oil-contaminated marine environments can degrade a wide range of crude oil components and under different redox conditions (Gauthier et al., 1992; Huu et al., 1999). It was also demonstrated that the degradation of crude oil components by *M. hydrocarbonoclasticus* SP17 involves the formation of oleolytic biofilms as an adaptation mechanism to degrade insoluble hydrocarbons (Mounier et al., 2014). Furthermore, *Marinobacter algicola* associated with dinoflagellate culture *Gymnodium catenatum* has previously been extensively investigated (Green et al., 2004). The presence of the members of genus *Marinobacter* in the initial cultures of *P. lutheri* and *N. oculata* suggests that these microbes are involved in the degradation of complex organic substrate produced by microalgae.

In our experiments with crude oil, a large number of sequences were also detected within the *Alcanivorax*, which is the most ubiquitous oil-degrading genus in marine environments (Yakimov et al., 1998, 2007). These sequences were affiliated to several alkane-degrading species, *Alcanivorax nanhaiticus* 19-m-6 (99% sequence identity), *Alcanivorax hongdengensis* A-11-3 (99%), and *Alcanivorax borkumensis* SK2 (98%; Supplementary Table S5). *Alcanivorax borkumensis* is an iconic cosmopolitan, naturally occurring, obligate hydrocarbonoclastic species playing a crucial role in the process of natural oil degradation (Yakimov et al., 1998; Harayama et al., 1999; Kasai et al., 2002; Schneiker et al., 2006; Reva et al., 2008). As described earlier, some species of genus *Alcanivorax* have been found in culture of the marine dinoflagellate *G. catenatum* (Green et al., 2004). Despite the wide distribution of hydrocarbonclastic bacteria of genera *Alcanivorax* and *Marinobacter*, their coexistence with microalgae remains largely unassessed. Our analysis of the geographic distribution of sites of *Alcanivorax* and *Marinobacter* isolation (or their molecular detection) in relation to the distribution of microalgae *Pavlova* and *Nannochloropsis* (Figure 5) suggests *Alcanivorax* and *Marinobacter* spp. in many cases were linked with the sites of isolation of *Pavlova* and *Nannochloropsis*. There is a much stronger (historically predetermined) effort being undertaken to map bacterial microbial diversity, whereas the isolation of phytoplankton is more tedious and of a lower throughput (Gilbert et al., 2014; Kopf et al., 2015; Thompson et al., 2017a), which is reflected in the larger coverage of bacteria, including *Alcanivorax* and *Marinobacter*. Unfortunately, it was not possible to prove the significant relationships of hydrocarbonoclastic bacteria and these microalgal cultures since the microalgal isolation is typically not accompanied by bacterial bioprospecting studies. The present study was also focused on two particular microalgal species. Other microalgal species, that were not evaluated here, may potentially also be natural hosts for hydrocarbon-degrading bacteria; however, an experimental assessment of this ability is still due. However, despite these limitations, our results represent an initial step toward improving our knowledge about associations of microalgae and oil-degrading bacteria and which could serve as a basis for future studies.

The less abundant sequences were derived from the two genera *Cobetia* and *Halomonas*. The sequences belonging to the *Cobetia* were enriched in NO samples. Most of the sequences were affiliated to *Cobetia amphilecti* 46-2 (100% sequence identity), *Cobetia litoralis* KMM 3880 (100%) and *Cobetia pacifica* KMM 3879 (100%). The representatives of this genus as previously reported by Al-Awadhi et al. (2012) were found in samples of coastal waters of Kuwait that were contaminated with oil. Pure cultures of members of genus *Cobetia* isolated from these samples produced biosurfactant.

In contrast to NO samples, the genus *Halomonas* was enriched in PO samples and the sequences related to this genus shared 99% similarity to *Halomonas lutescens* Q1U (99.6% sequence identity) and *Halomonas zhaodongensis* NEAU-ST10-25 (99.6%). The members of genus *Halomonas* were detected in the samples of the deep-water plume during the DWH spill (Gutierrez et al., 2013). It has been reported that *Halomonas* species can degrade aromatic and aliphatic hydrocarbons indicating their hydrocarbon-degrading potential (Pepi et al., 2005; Cui et al., 2008; Mnif et al., 2009).

Alphaproteobacteria that were enriched with oil in our experiments in NO samples included members of the genera *Roseovarius*, *Hyphomonas* and *Tropicibacter*. The representatives of the *Roseovarius* within the Roseobacter clade have been previously reported as a common genus in oil-contaminated environments (Kimes et al., 2013; Kappell et al., 2014). The members of this clade have been also found in marine communities from oceanic phytoplankton assemblages (Gonzalez et al., 2000). The members of genus *Hyphomonas* have been detected previously in both mesocosms and microcosm enrichments with oil, suggesting their involvement in the hydrocarbon degradation (Yakimov et al., 2005; Coulon et al., 2007; Kappell et al., 2014). Moreover, in our study, several detected sequencing reads were also related to the genus *Tropicibacter* (Supplementary Table S5). It has been shown previously that some species of this genus, for example, *T. naphthalenivorans*, are capable of degrading polycyclic aromatic hydrocarbons (Harwati et al., 2009).

The hydrocarbon-degrading members of the phylum Bacteroidetes are often detected in marine communities from contaminated sites (Beazley et al., 2012). They are also found to be associated with phytoplankton species (O’Sullivan et al., 2004). Our results showed that many sequences of the phylum Bacteroidetes detected in our samples were affiliated to the Flavobacteriaceae and Cryomorphaceae. Among these families, the genera *Owenweeksia*, *Muricauda*, and *Marivirga* were enriched in our experiments on the addition of crude oil. The phylogenetic affiliation of sequences within these genera showed a high similarity matching with the strains *Owenweeksia hongkongensis* UST20020801 (92% identity), *Muricauda indica* 3PC125-7 (100% identity), and *Muricauda marina* H19-56 (100% identity; Supplementary Table S5).

Genera that can degrade aromatic hydrocarbons were identified in our enrichments as *Polycyclovorans* (class Gammaproteobacteria, family Xanthomonadales) and *Arenibacter* (class Bacteroidetes, family Flavobacteriaceae). Their sequences had 95.28% identity to *Polyciclovorance algicola* and 100% identity to *Arenibacter algicola* TG409 (Supplementary Table S5). These strains have been recently isolated from marine phytoplankton cultures by Gutierrez et al. (2012a, 2013) who demonstrated the ability of these organisms to degrade aromatic substrates. Although the proportion of the community capable of oil-utilization as a function of total diversity has not been assessed in this study; however, the abundance of enriched taxa strongly suggests they play a significant role in the degradation of oil hydrocarbons.

We also performed sequencing of the full-length of the 16S rRNA gene using the Oxford Nanopore platform. While the Illumina short reads offered the accuracy and depth of sequences, the length of the sequencing reads is considered as a limiting factor in the analysis of complex bacterial communities, particularly at the genus level (Shin et al., 2018). In this case, the full-length sequencing reads of the 16S rRNA gene are considered more advantageous in the determination of taxa at a fine taxonomical level, such as genus, though with the only limitation being the high error rate in the case of Nanopore sequencing. The bacterial profile from the oil-enriched microbial communities at the genus level obtained with Nanopore data also confirmed the sequencing data we obtained with the Illumina platform. This similarity in sequencing data obtained by both methods is based on the proportion of main genera in the bacterial communities. However, in contrast to the Illumina data, the results obtained with full-length sequencing reads using Nanopore allowed us to detect more genera in the oil enrichments of both microalgae species (Figure 2, Supplementary Table S4).

Our attempts to isolate and identify the hydrocarbon-degrading microorganisms from the enrichments with crude oil by using a culture-dependent method resulted in the isolation of the total 48 bacterial strains (Figure 4, Supplementary Table S7). A comparison of the bacterial genera identified by 16S rRNA genes in oil enrichments with the obtained isolates revealed conformity for *P. lutheri* and *N. oculata*. The sequencing analysis of these isolates showed that most of them belonged to the Proteobacteria (classes Alpha- and Gamma-proteobacteria) and Bacteroidetes. For example, in the enrichment of *P. lutheri*, the isolates related to genus *Marinobacter* (28% of strains isolated) and genus *Alcanivorax* (12% of strains isolated) were identified in agreement with the Illumina analysis, where these genera represented about 35 and 12% of the bacterial population, respectively (Supplementary Table S3). In the cases of isolates obtained from the enrichment of *N. oculata*, the number of strains belonging to Alpha- and Gamma-proteobacteria were almost equal (representing 32 and 44% of total isolates). The Gammaproteobacteria were dominated by isolated species of the *Alcanivorax*, the main genus identified by the Illumina analysis and *Oleibacter* that was identified as a minor fraction in enrichment community by the same analysis. The relative abundance of *Marinobacter* sequences was high in NO enrichment cultures and representatives of this genus are, in general, easy to cultivate; however, we obtained only one non-redundant isolate of *Marinobacter* spp., which may point at the predominance of this isolate in enrichment culture. The presence of *Marinobacter* and *Alcanivorax* species associated with microalgae detected both by culture-independent analysis and cultivation approach suggested their importance not only as known hydrocarbon degraders but also as species supporting the growth of microalgae in general. The bacterial strains isolated from the enrichments of both *P. lutheri* and *N. oculata* and belonging to the phylum Bacteroidetes were identified as the species of genera *Marivirga* and *Roseivirga* (enrichment of *P. lutheri*) and species of genus *Maribacter* (enrichment of *N. oculata*; Supplementary Table S7).

Our results showed that microalgal bioreactor cultures of *P. lutheri* and *N. oculata* hosted diverse communities of microorganisms including specific groups (hydrocarbonoclastic bacteria and hydrocarbon-degrading bacteria) of organisms that are able to degrade hydrocarbons. As a response to the crude oil supplementation, there was a strong selection for “classical” hydrocarbon-degrading bacteria of genera *Alcanivorax* and *Marinobacter*. Both were detectable at extremely low levels (<0.5%) in the microalgal cultures but became predominant (>50%) in crude oil-enriched consortia. Isolation of a few dozen hydrocarbon-degrading bacteria of both predominant genera has complemented the culture-independent analysis. There is generally a lack in our understanding of natural niches and sources of hydrocarbon-degrading specialist bacteria, previously commonly attributed to technogenic oil spills and natural seeps. Our study suggests the potential importance of these microalgal species as natural reservoirs for oil-degrading bacterial groups of organisms.

## Data Availability Statement

The datasets presented in this study can be found in online repositories. The names of the repository/repositories and accession number(s) can be found in the article/Supplementary Material.

## Author Contributions

Conceptualization: TC, MY, DT, and PG. Methodology: TC. Investigation: TC, VS, and EL. Resources: PG and DT. Supervision: PG. Data curation: RB and ST. Writing-original draft preparation: TC. Writing-review and editing: TC, RB, ST, VS, EL, DT, MY, and PG. All authors have read and agreed to the published version of the manuscript.

### Conflict of Interest

The authors declare that the research was conducted in the absence of any commercial or financial relationships that could be construed as a potential conflict of interest.

## Footnotes

^1^https://github.com/jstjohn/SeqPrep
