# Supplementary material for: Hydrocarbon-Degrading Bacteria Alcanivorax and Marinobacter Associated With Microalgae Pavlova lutheri and Nannochloropsis oculata
Source: Front Microbiol. 2020 Oct 28;11:572931. doi: 10.3389/fmicb.2020.572931 (PMC7655873; doi:10.3389/fmicb.2020.572931)
Supplement: Supplementary file 1 [file Data_Sheet_1.zip › Suppl_files_final/Supplementary_file_01.10.2020.docx]

**Supplementary Materials**

**Figure S1.** Rarefaction plots of diversity in microalgae-associated bacterial communities in all experiments (A-Illumina data; B-Nanopore data).

Solid lines showed the numbers of OTUs obtained in all experimental samples. Dashed lines showed the predicted numbers of OTUs in each experimental sample.

N, the culture of *Nannochloropsis oculata*; NO, enrichment of *N. oculata* culture with crude oil; ECN, control of *N. oculata* culture without crude oil; P, the culture of *Pavlova lutheri*; PO, enrichment of *P. lutheri* culture with crude oil; ECP, control of. P*. lutheri* culture without crude oil.

**A B**


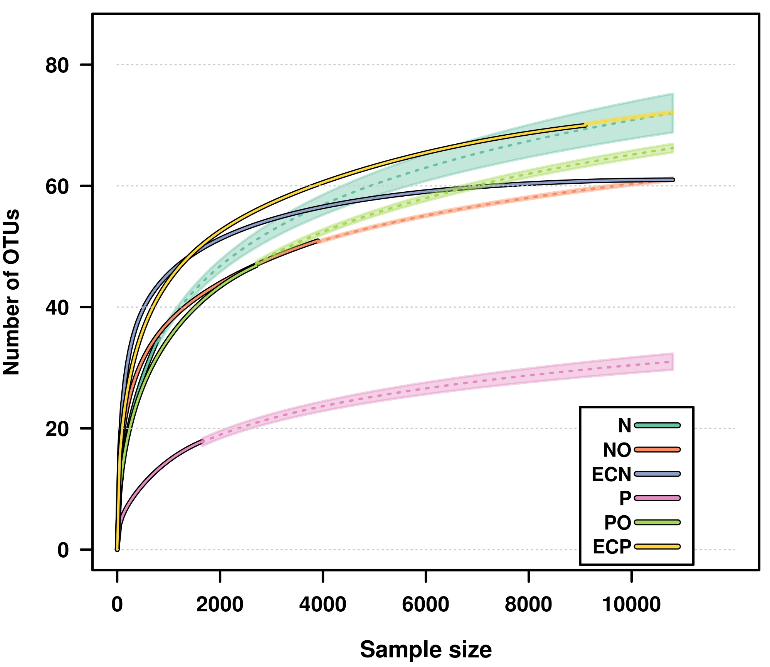

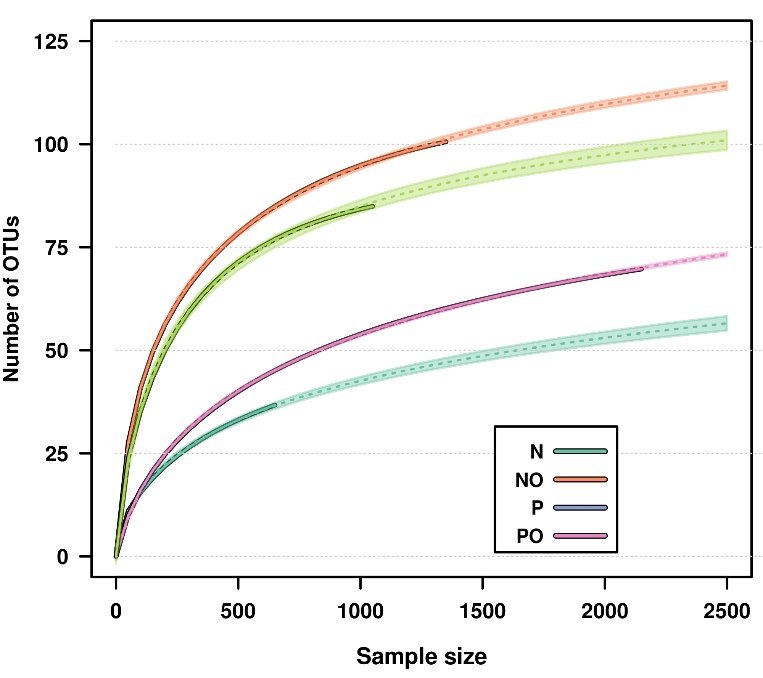


**Table S1.** Comparisons of taxonomic distribution of reads at Phylum level between P, PO, N, NO, ECN and ECP samples (Illumina data).

| **Phylum** |  | | | | | |
| --- | --- | --- | --- | --- | --- | --- |
|  | **N** | **NO** | **ECN** | **P** | **PO** | **ECP** |
| *Proteobacteria* | 80.69307 | 91.12018 | 77.21661 | 86.63594 | 93.17932 | 60.86045 |
| *Acidobacteria* | 0 | 0.025517 | 0 | 0 | 0 | 0 |
| *Actinobacteria* | 0 | 0 | 0.018706 | 0 | 0.03667 | 0.011031 |
| *Bacteroidetes* | 8.415842 | 4.618525 | 18.05088 | 11.98157 | 2.090209 | 7.545505 |
| *Chlorobi* | 0 | 0 | 0 | 0 | 0 | 0.121346 |
| *Chloroflexi* | 0 | 0 | 0 | 0 | 0 | 0.242692 |
| *Deinococcus-Thermus* | 0.49505 | 0 | 0 | 0.460829 | 0 | 0 |
| *Firmicutes* | 0.49505 | 0.1531 | 0.074822 | 0 | 0 | 14.00993 |
| *Nitrospirae* | 0 | 0 | 0 | 0 | 0.03667 | 0 |
| *Planctomycetes* | 9.90099 | 3.546823 | 3.647587 | 0 | 4.583792 | 9.167126 |
| *Verrucomicrobia* | 0 | 0.535851 | 0.897868 | 0 | 0.073341 | 0 |
| Unassigned | 0 | 0 | 0.093528 | 0.921659 | 0 | 8.041919 |

**Table S2.** Comparisons of taxonomic distribution of reads at Class level between P, PO, N, NO, ECN and ECP samples (Illumina data).

| **CLASS** |  | | | | | |
| --- | --- | --- | --- | --- | --- | --- |
|  | **N** | **NO** | **ECN** | **P** | **PO** | **ECP** |
| *α-Proteobacteria* | 75.24752 | 22.48022 | 54.90086 | 86.17512 | 42.61093 | 36.90017 |
| *β-Proteobacteria* | 0 | 0.051033 | 0.018706 | 0 | 0 | 0.07722 |
| *γ-Proteobacteria* | 4.455446 | 66.39449 | 22.03517 | 0.460829 | 50.3117 | 23.861 |
| *δ-Proteobacteria* | 0.990099 | 2.194437 | 0.261878 | 0 | 0.256692 | 0.022063 |
| *Acidobacteria* | 0 | 0.025517 | 0 | 0 | 0 | 0 |
| *Actinobacteria* | 0 | 0 | 0.018706 | 0 | 0.03667 | 0.011031 |
| *Rhodothermi* | 0 | 0 | 4.115226 | 0 | 0 | 0.23166 |
| *Saprospirae* | 0 | 0 | 0 | 0 | 0 | 0.143409 |
| *Bacteroidia* | 0.49505 | 0 | 0 | 0 | 0 | 0 |
| *Cytophagia* | 2.475248 | 0.433784 | 11.24205 | 0 | 1.06344 | 6.674021 |
| *Flavobacteriia* | 2.475248 | 4.108191 | 2.693603 | 11.98157 | 0.916758 | 0.165472 |
| *Sphingobacteriia* | 2.970297 | 0 | 0 | 0 | 0 | 0.330943 |
| *Other Bacteroidetes* | 0 | 0.07655 | 0 | 0 | 0.110011 | 0 |
| *Chlorobi* | 0 | 0 | 0 | 0 | 0 | 0.121346 |
| *Anaerolineae* | 0 | 0 | 0 | 0 | 0 | 0.242692 |
| *Deinococci* | 0.49505 | 0 | 0 | 0.460829 | 0 | 0 |
| *Bacilli* | 0 | 0 | 0.056117 | 0 | 0 | 14.00993 |
| *Clostridia* | 0.49505 | 0.1531 | 0.018706 | 0 | 0 | 0 |
| *Nitrospira* | 0 | 0 | 0 | 0 | 0.03667 | 0 |
| *Phycisphaerae* | 0 | 0.1531 | 0 | 0 | 4.40044 | 9.167126 |
| *Planctomycetacia* | 9.90099 | 0.739985 | 0 | 0 | 0.03667 | 0 |
| *Planctomycetia* | 0 | 0 | 0.392817 | 0 | 0 | 0 |
| *Other Planctomycetes* | 0 | 2.653738 | 3.25477 | 0 | 0.146681 | 0 |
| *Opitutae* | 0 | 0.1531 | 0 | 0 | 0 | 0 |
| *Verrucomicrobiae* | 0 | 0.382751 | 0.897868 | 0 | 0.073341 | 0 |
| Unassigned | 0 | 0 | 0.093528 | 0.921659 | 0 | 8.041919 |

**Table S3.** Comparisons of taxonomic distribution of reads at Genus level between P, PO, N, NO, ECN and ECP samples (Illumina data).

| **GENUS** |  | | | | | |
| --- | --- | --- | --- | --- | --- | --- |
|  | **N** | **NO** | **ECN** | **P** | **PO** | **ECP** |
| *Marinobacter* | 0.49505 | 23.67951 | 0.374111 | 0.460829 | 34.94683 | 0.375069 |
| *Alcanivorax* | 0.49505 | 28.7063 | 0.523756 | 0 | 12.65127 | 3.011583 |
| *Hyphomonas* | 0 | 1.454453 | 0.617284 | 0 | 7.884122 | 0.672918 |
| *Cobetia* | 0 | 5.256443 | 0 | 0 | 0.146681 | 0 |
| *Oceanicaulis* | 7.920792 | 3.036489 | 11.44781 | 0 | 0.073341 | 2.879206 |
| *Rhodopirellula* | 9.90099 | 0.739985 | 0 | 0 | 0.03667 | 0 |
| *Oricola* | 12.87129 | 0.025517 | 0 | 0 | 0 | 0 |
| *Roseovarius* | 1.980198 | 2.066854 | 0 | 0.460829 | 0.366703 | 0 |
| *Halomonas* | 0.49505 | 0.255167 | 0.617284 | 0 | 1.943528 | 0.30888 |
| *Roseivirga* | 0 | 0.025517 | 10.92406 | 0 | 0.256692 | 6.66299 |
| *Ulvibacter* | 0 | 0 | 0 | 10.59908 | 0.03667 | 0 |
| *Bacillus* | 0 | 0 | 0.037411 | 0 | 0 | 13.88858 |
| *Hoeflea* | 4.455446 | 0 | 0 | 0 | 0 | 0 |
| *Tropicibacter* | 8.910891 | 7.272263 | 0 | 54.37788 | 28.96956 | 0 |
| *Thalassospira* | 0 | 0 | 0 | 0.460829 | 0.03667 | 4.500827 |
| *Labrenzia* | 0 | 0 | 0 | 4.608295 | 0.073341 | 0 |
| *Balneola* | 0 | 0.07655 | 4.115226 | 0 | 0.110011 | 0.23166 |
| *Devosia* | 0.990099 | 0.204134 | 0.299289 | 0 | 0 | 2.846111 |
| *Sulfitobacter* | 3.960396 | 0.051033 | 0.056117 | 0 | 0 | 0 |
| *Alteromonas* | 0 | 0 | 2.91807 | 0 | 0 | 1.025924 |
| Other Bacteria | 37.62376 | 24.01123 | 67.97606 | 25.80645 | 11.22112 | 55.55433 |
| uncultured | 9.90099 | 3.087522 | 0 | 1.843318 | 1.026769 | 0 |
| Ambiguous_taxa | 0 | 0.051033 | 0 | 0.460829 | 0.220022 | 0 |
| Unassigned | 0 | 0 | 0.093528 | 0.921659 | 0 | 8.041919 |
|  |  |  |  |  |  |  |
|  |  |  |  |  |  |  |
|  |  |  |  |  |  |  |
| **Other Bacteria** |  |  |  |  |  |  |
| HTCC | 0 | 0 | 5.031799 | 0 | 0 | 18.01434 |
| *Owenweeksia* | 0 | 3.444756 | 0 | 0 | 0 | 0 |
| *OM27clade* | 0.990099 | 2.194437 | 0 | 0 | 0.256692 | 0 |
| *Muricauda* | 0.49505 | 0.306201 | 0.018706 | 1.382488 | 0.696736 | 0.011031 |
| *Cyclobacterium* | 2.475248 | 0.408267 | 0 | 0 | 0 | 0 |
| *Loktanella* | 0 | 0 | 2.151141 | 0 | 0 | 0.110314 |
| *Thalassobaculum* | 0 | 0.025517 | 0 | 1.382488 | 0.660066 | 0 |
| *Kordia* | 1.980198 | 0.025517 | 0 | 0 | 0 | 0 |
| *Salegentibacter* | 0 | 0 | 1.851852 | 0 | 0 | 0.011031 |
| *Paracoccus* | 0.49505 | 1.020669 | 0.018706 | 0 | 0.073341 | 0.022063 |
| *Phyllobacterium* | 0.49505 | 0.127584 | 0 | 0.921659 | 0.073341 | 0 |
| *Ruegeria* | 1.485149 | 0.051033 | 0 | 0 | 0 | 0 |
| *Litorimonas* | 0 | 0.433784 | 0 | 0.921659 | 0.073341 | 0 |
| *Novosphingobium* | 0 | 0 | 0.037411 | 0 | 0 | 1.180364 |
| *Maritalea* | 0 | 0.025517 | 0 | 0.921659 | 0.073341 | 0 |
| *Nitratireductor* | 0 | 0 | 0.074822 | 0 | 0.770077 | 0.165472 |
| *Metallibacterium* | 0.990099 | 0 | 0 | 0 | 0 | 0 |
| *Methylophaga* | 0 | 0 | 0.97269 | 0 | 0 | 0 |
| *Thermus* | 0.49505 | 0 | 0 | 0.460829 | 0 | 0 |
| *Verrucomicrobium* | 0 | 0 | 0.897868 | 0 | 0 | 0 |
| *Marivirga* | 0 | 0 | 0 | 0 | 0.806747 | 0 |
| *Arenibacter* | 0 | 0.255167 | 0.486345 | 0 | 0.03667 | 0 |
| *Pseudoalteromonas* | 0.49505 | 0 | 0.112233 | 0 | 0 | 0 |
| *Bacteroides* | 0.49505 | 0 | 0 | 0 | 0 | 0 |
| *Marinomonas* | 0.49505 | 0 | 0 | 0 | 0 | 0 |
| *Acidibacter* | 0.49505 | 0 | 0 | 0 | 0 | 0 |
| *Hyphomicrobium* | 0 | 0 | 0.467639 | 0 | 0 | 0 |
| *Altererythrobacter* | 0 | 0.408267 | 0 | 0 | 0 | 0 |
| *Polycyclovorans* | 0 | 0.22965 | 0 | 0 | 0.073341 | 0 |
| *Maricaulis* | 0 | 0 | 0 | 0 | 0 | 0.15444 |
| *Cerasicoccus* | 0 | 0.1531 | 0 | 0 | 0 | 0 |
| *Costertonia* | 0 | 0 | 0 | 0 | 0.146681 | 0 |
| *Pseudohongiella* | 0 | 0.051033 | 0 | 0 | 0.073341 | 0 |
| *Candidatus Portiera* | 0 | 0 | 0 | 0 | 0 | 0.110314 |
| *Kangiella* | 0 | 0 | 0 | 0 | 0.110011 | 0 |
| *Haliea* | 0 | 0.07655 | 0 | 0 | 0 | 0 |
| *Amorphus* | 0 | 0 | 0 | 0 | 0.073341 | 0 |
| *Oleibacter* | 0 | 0.051033 | 0 | 0 | 0 | 0.022063 |
| *Idiomarina* | 0 | 0 | 0.056117 | 0 | 0 | 0 |
| *Leptospirillum* | 0 | 0 | 0 | 0 | 0.03667 | 0 |
| *Methylobacterium* | 0 | 0 | 0 | 0 | 0.03667 | 0 |
| *Porphyrobacter* | 0 | 0 | 0 | 0 | 0.03667 | 0 |
| *Ralstonia* | 0 | 0.025517 | 0 | 0 | 0 | 0 |
| *Aquabacterium* | 0 | 0.025517 | 0 | 0 | 0 | 0 |
| *Paenibacillus* | 0 | 0 | 0 | 0 | 0 | 0.022063 |
| *Congregibacter* | 0 | 0 | 0 | 0 | 0 | 0.022063 |
| *Micrococcus* | 0 | 0 | 0.018706 | 0 | 0 | 0 |
| *Agrobacterium* | 0 | 0 | 0.018706 | 0 | 0 | 0 |
| *Anaerospora* | 0 | 0 | 0.018706 | 0 | 0 | 0 |
| *Rhodococcus* | 0 | 0 | 0 | 0 | 0 | 0.011031 |
| *Streptococcus* | 0 | 0 | 0 | 0 | 0 | 0.011031 |
| *Rhizobium* | 0 | 0 | 0 | 0 | 0 | 0.011031 |
| *Haererehalobacter* | 0 | 0 | 0 | 0 | 0 | 0.011031 |
| *C1-B045* | 0.49505 | 8.063281 | 0 | 0 | 0.366703 | 0 |
| *SM1A02* | 0 | 0 | 0 | 0 | 4.070407 | 0 |
| *Phycisphaera* | 0 | 0 | 0 | 0 | 0.330033 | 0 |
| *Algisphaera* | 0 | 0.1531 | 0 | 0 | 0 | 0 |
| Other Bacteria | 25.24752 | 6.455729 | 55.74261 | 19.81567 | 2.346901 | 35.66464 |

**Table S4**. The relative abundance and distribution of the reads assigned to Genus level in the samples NO and PO by Illumina and Nanopore.

|  | **Illumina** | | **Nanopore** | |
| --- | --- | --- | --- | --- |
|  | **NO** | **PO** | **NO** | **PO** |
| *Roseivirga* | 0.02551671 | 0.25669234 | 0.07 | 0.48 |
| *Cyclobacterium* | 0.40826742 | 0 | 0.58 | 0.00 |
| *Other Cytophagales* | 0 | 0.80674734 | 0.22 | 0.86 |
| *Muricauda* | 0.30620056 | 0.69673634 | 0.51 | 0.67 |
| ***Other Flavobacteriales*** | 3.8019903 | 0.220022 | 3.07 | 0.00 |
| *Balneola* | 0.07655014 | 0.110011 | 0 | 0 |
| *Rhodopirellula* | 0.73998469 | 0.03667033 | 2.48 | 0.00 |
| *Phycisphaerales* | 0.15310028 | 4.40044004 | 0.00 | 12.08 |
| *Other Planctomycetes* | 2.6537382 | 0.14668133 | 2.78 | 0.00 |
| *Tropicibacter* | 7.27226333 | 28.9695636 | 0 | 0 |
| *Oceanicaulis* | 3.0364889 | 0.07334067 | 0 | 0 |
| *Roseovarius* | 2.06685379 | 0.36670334 | 4.24 | 2.66 |
| *Sulfitobacter* | 0.05103343 | 0 | 1.39 | 2.95 |
| *Loktanella* | 0 | 0 | 0.29 | 1.43 |
| *Marinovum* | 0 | 0 | 0.00 | 1.90 |
| *Ruegeria* | 0 | 0 | 0.15 | 2.76 |
| *Sediminimonas* | 0 | 0 | 0.22 | 5.04 |
| *Tateyamaria* | 0 | 0 | 0.88 | 0.76 |
| *Thalassobius* | 0 | 0 | 0.22 | 0.86 |
| ***Other Rhodobacterales*** | 5.33299311 | 2.89695636 | 13.95 | 27.59 |
| ***Rhizobiales*** | 1.3268691 | 1.02676934 | 0.07 | 0.57 |
| *Hyphomonas* | 1.45445267 | 7.88412175 | 2.63 | 7.99 |
| *Other Caulobacterales* | 0.43378413 | 0.14668133 | 2.92 | 1.14 |
| ***Other α-Proteobacteria*** | 1.50548609 | 1.24679135 | 4.09 | 0.10 |
| *β-Proteobacteria* | 0.05103343 | 0 | 0 | 0 |
| *Marinobacter* | 23.6795101 | 34.946828 | 21.62 | 22.93 |
| *Cellvibrionales* | 8.13983159 | 0.36670334 | 7.60 | 0.10 |
| *Alcanivorax* | 28.7063026 | 12.6512651 | 16.80 | 6.76 |
| *Cobetia* | 5.25644297 | 0.14668133 | 2.85 | 0.00 |
| *Oceanospirillales* | 0.35723399 | 2.12687935 | 0.44 | 0.19 |
| *Other γ-Proteobacteria* | 0.25516713 | 0.07334067 | 1.53 | 0.19 |
| *δ-Proteobacteria* | 2.19443736 | 0.25669234 | 4.38 | 0.00 |
| *Acidobacteria* | 0.02551671 | 0 | 0 | 0 |
| *Actinobacteria* | 0 | 0.03667033 | 0 | 0 |
| *Firmicutes* | 0.15310028 | 0 | 0 | 0 |
| *Verrucomicrobia* | 0.53585098 | 0.07334067 | 4.02 | 0.00 |
| *Other Bacteria* | 0 | 0.03667033 | 0 | 0 |

**Table S5**. The list of the most abundant Illumina sequences obtained in the oil enrichments PO and NO and their closets matches in GenBank.

| **Phylum** | **Class** | **Closest organism** | **GenBank accession number** | **% similarity** |
| --- | --- | --- | --- | --- |
| *Proteobacteria* | *Gammaproteobacteria* | *Marinobacter hydrocarbonoclasticus* VT8 | NR_027551.1 | 100 |
|  |  | *Marinobacter hydrocarbonoclasticus* SP17 | NR_074619.1 | 100 |
|  |  | *Marinobacter algicola* DG893 | NR_042807.1 | 100 |
|  |  | *Alcanivorax nanhaiticus* 19-m-6 | NR_152008.1 | 99.6 |
|  |  | *Alcanivorax hongdengensis* A-11-3 | NR_044499.1 | 99.2 |
|  |  | *Alcanivorax borkumensis* SK2 | NR_074890.1 | 98.42 |
|  |  | *Alcanivorax gelatiniphagus* | NR_136483.1 | 97.23 |
|  |  | *Cobetia amphilecti* 46-2 | NR_113404.1 | 100 |
|  |  | *Cobetia litoralis* KMM 3880 | NR_113403.1 | 100 |
|  |  | *Cobetia pacifica* KMM 3879 | NR_113402.1 | 100 |
|  |  | *Polyciclovorance algicola* | NR_116560.1 | 95.28 |
|  | *Alphaproteobacteria* | *Roseovarius nubinhibens* ISM | NR_028728.1 | 99.6 |
|  |  | *Halomonas lutescens* Q1U | NR_152713.1 | 99.6 |
|  |  | *Halomonas zhaodongensis* NEAU-ST10-25 | NR_125612.1 | 99.6 |
|  |  | *Hyphomonas beringensis* MCCC 1A07321 | NR_148267.1 | 98.81 |
|  |  | *Hyphomonas chukchiensis* MCCC 1A07481 | NR_148268.1 | 98.42 |
|  |  | *Tropicibacter multivorans* MD5T | NR_108509.1 | 100 |
| *Bacteroidetes* | *Flavobacteriia* | *Owenweeksia hongkongensis* UST20020801 | NR_040990.1 | 92.13 |
|  |  | *Arenibacter algicola* TG409 | NR_116561.1 | 100 |
|  |  | *Muricauda indica* 3PC125-7 | NR_159923.1 | 100 |
|  |  | *Muricauda marina* H19-56 | NR_157633.1 | 100 |
| *Planctomycetes* | *Planctomycetacia* | *Rhodopirellula* *baltica* SH1 | NR_043384.1 | 100 |
|  |  | *Rhodopirellula* *rubra* LF2 | NR_126223.1 | 100 |
|  | *Phycisphaerae* | *Algisphaera agarilytica* 06SJR6-2 | NR_125472.1 | 97.63 |

**Table S6.** The taxonomic abundances of OTUs from P, PO, N, NO, ECN, and ECP samples (Illumina data) used for non-metric multidimensional analysis (NMDS) and statistical analyses using the General linear model (GLM) and Likelihood ratio test (LRT).

|  | **N** | **NO** | **ECN** | **P** | **PO** | **ECP** |
| --- | --- | --- | --- | --- | --- | --- |
| *Acidobacteria* | 0 | 0.02551671 | 0 | 0 | 0 | 0 |
| *Actinobacteria* | 0 | 0 | 0.01870557 | 0 | 0.03667033 | 0.01103144 |
| *Balneola* | 0 | 0 | 4.11522634 | 0 | 0 | 0.23166023 |
| *Roseivirga* | 0 | 0.02551671 | 10.9240554 | 0 | 0.25669234 | 6.66298952 |
| *Ulvibacter* | 0 | 0 | 0 | 10.5990783 | 0.03667033 | 0 |
| *Other Flavobacteriales* | 2.47524752 | 4.10819087 | 2.69360269 | 1.38248848 | 0.88008801 | 0.16547159 |
| *Other Bacteroidetes* | 5.94059406 | 0.48481756 | 0.31799476 | 0 | 0.91675834 | 0.48538334 |
| *Bacillus* | 0 | 0 | 0.03741115 | 0 | 0 | 13.8885825 |
| *Other Firmicutes* | 0.4950495 | 0.15310028 | 0.03741115 | 0 | 0 | 0.12134584 |
| *Rhodopirellula* | 9.9009901 | 0.73998469 | 0 | 0 | 0.03667033 | 0 |
| *Phycisphaerales* | 0 | 0.15310028 | 0 | 0 | 4.40044004 | 9.16712631 |
| *Planctomycetes* | 0 | 2.6537382 | 3.64758698 | 0 | 0.14668133 | 0 |
| *Sphingomonadales* | 0 | 0.40826742 | 2.02020202 | 0 | 0.03667033 | 2.07391065 |
| *Rhodospirillales* | 0.4950495 | 0.02551671 | 0.87916199 | 1.84331797 | 0.69673634 | 9.63044677 |
| *Tropicibacter* | 8.91089109 | 7.27226333 | 0 | 54.3778802 | 28.9695636 | 0 |
| *Oceanicaulis* | 7.92079208 | 3.0364889 | 11.4478114 | 0 | 0.07334067 | 2.87920574 |
| *Roseovarius* | 1.98019802 | 2.06685379 | 0 | 0.46082949 | 0.36670334 | 0 |
| *Labrenzia* | 0 | 0 | 0 | 4.60829493 | 0.07334067 | 0 |
| *Sulfitobacter* | 3.96039604 | 0.05103343 | 0.05611672 | 0 | 0 | 0 |
| *Other Rhodobacterales* | 27.2277228 | 5.33299311 | 36.9248036 | 20.7373272 | 2.82361569 | 11.7926089 |
| *Oricola* | 12.8712871 | 0.02551671 | 0 | 0 | 0 | 0 |
| *Hoeflea* | 4.45544554 | 0 | 0 | 0 | 0 | 0 |
| *Devosia* | 0.99009901 | 0.20413371 | 0.29928919 | 0 | 0 | 2.84611142 |
| *Other Rhizobiales* | 6.43564356 | 1.09721868 | 0.93527871 | 1.84331797 | 1.02676934 | 2.25041368 |
| *Hyphomonas* | 0 | 1.45445267 | 0 | 0 | 7.88412175 | 0 |
| *Other Caulobacterales* | 0 | 0.43378413 | 0 | 2.30414747 | 0.14668133 | 0 |
| *Thalassospira* | 0 | 0 | 0 | 0 | 0 | 4.50082736 |
| *Other α-Proteobacteria* | 0 | 1.07170196 | 2.33819678 | 0 | 0.51338467 | 0.92664093 |
| *β-Proteobacteria* | 0 | 0.05103343 | 0.01870557 | 0 | 0 | 0.07722008 |
| *Marinobacter* | 0.4950495 | 23.6795101 | 0.37411149 | 0.46082949 | 34.946828 | 0.37506895 |
| *HTCC* | 0 | 0 | 5.03179948 | 0 | 0 | 18.0143409 |
| *Other Alteromonadales* | 0.4950495 | 0 | 5.85484474 | 0 | 0 | 1.2244898 |
| *Cellvibrionales* | 0.4950495 | 8.13983159 | 0 | 0 | 0.36670334 | 0 |
| *Alcanivorax* | 0.4950495 | 28.7063026 | 0.52375608 | 0 | 12.6512651 | 3.01158301 |
| *Cobetia* | 0 | 5.25644297 | 0 | 0 | 0.14668133 | 0 |
| *Oceanospirillales* | 0.99009901 | 0.35723399 | 1.53385709 | 0 | 2.12687935 | 0.5846663 |
| *Xanthomonadales* | 1.48514851 | 0.25516713 | 6.64047886 | 0 | 0.07334067 | 0.14340871 |
| *Other γ-Proteobacteria* | 0 | 0 | 2.07631874 | 0 | 0 | 0.50744622 |
| *δ-Proteobacteria* | 0.99009901 | 2.19443736 | 0.26187804 | 0 | 0.25669234 | 0.02206288 |
| *Other Bacteria* | 0.4950495 | 0.53585098 | 0.89786756 | 0.46082949 | 0.110011 | 0.36403751 |
| Unassigned | 0 | 0 | 0.09352787 | 0.92165899 | 0 | 8.04191947 |

**Table S7.** The list of isolates obtained from the enrichments of PO (*Pavlova lutheri*) and NO (*Nanochloropsis oculata*) and their closest organisms in the NCBI database.

| Isolate abbreviation | Closest relative | NCBI Ref Seq | Sequence Identity  % |
| --- | --- | --- | --- |
|  | ***Pavlova lutheri*** |  |  |
| P1  P2  P3  P4  P5  P6  P7  P9  P10  P11  P12  P14  P15  P16  P17  P18  P19  P20  P21  P22  P23  P24  P25  P26  P28 | *Marinobacter algicola*  *Marivirga tractuosa*  *Marinobacter algicola*  *Marinovum algicola*  *Halomonas meridiana*  *Thalassospira tepidiphila*  *Alcanivorax jadensis*  *Halomonas meridiana*  *Hyphomonas beringensis*  *Thalassospira tepidiphila*  *Marivirga tractuosa*  *Marinobacter algicola*  *Alcanivorax nanhaiticus*  *Marinobacter algicola*  *Marinobacter algicola*  *Marinobacter algicola*  *Hyphomonas beringensis*  *Alcanivorax jadensis*  *Hyphomonas beringensis*  *Roseivirga seohaensis*  *Thalassospira indica*  *Marivirga tractuosa*  *Marinovum algicola*  *Maritalea myrionectae*  *Marinobacter algicola* | NR_042807.1  NR_074493.1  NR_042807.1  NR_115896.1  NR_042066.1  NR_041492.1  NR_025271.1  NR_042066.1  NR_148267.1  NR_041492.1  NR_074493.1  NR_042807.1  NR_152008.1  NR_042807.1  NR_042807.1  NR_042807.1  NR_148267.1  NR_025271.1  NR_148267.1  NR_153705.1  NR_153721.1  NR_074493.1  NR_112651.1  NR_044360.1  NR_042807.1 | 99.03%  94.31%  99.2%  99.74%  98.6%  99.5%  97.35%  98.71%  98.73%  99.83%  94.44%  99.53%  96.66%  99.53%  99.59%  98.37%  99.22%  98.75%  99.24%  100%  99.62%  96.58%  100%  99.19%  99.32% |
|  | ***Nannochloropsis oculata*** |  |  |
| N1  N2  N3  N4  N5  N6  N7  N8  N9  N10  N11  N12  N13  N14  N16  N17  N18  N19  N21  N22  N23  N24  N25 | *Alcanivorax xenomutans*  *Alcanivorax borkumensis*  *Alcanivorax jadensis*  *Alcanivorax xenomutans*  *Marinobacter algicola*  *Paraglaciecola chathamensis*  *Alteromonas naphtalenivorans*  *Oleibacter marinus*  *Oleibacter marinus*  *Oleibacter marinus*  *Oceanobacter kriegii*  *Cobetia amphilecti*  *Thalassospira tepidiphila*  *Albimonas donghaensis*  *Litorimonas haliclonae*  *Boseongicola aestuarii*  *Boseongicola aestuarii*  *Tropicibacter naphthalenivorans*  *Litorimonas haliclonae*  *Maribacter dokdonensis*  *Kordia algicida*  *Balneola alkaliphila*  *Balneola alkaliphila* | NR_133958.1  NR_074890.1  NR_025271.1  NR_133958.1  NR_042807.1  NR_041397.1  NR_145589.1  NR_114287.1  NR_114287.1  NR_114287.1  NR_024655.1  NR_113404.1  NR_041492.1  NR_043685.1  NR_159917.1  NR_133983.1  NR_133983.1  NR_041596.1  NR_159917.1  NR_043294.1  NR_027568.1  NR_044367.1  NR_044367.1 | 99.9%  99.52%  98.21%  98.46%  99.84%  99.43%  98.45%  98.19%  98.11%  98.11%  96.57%  100%  100%  99.76%  98.89%  97.36%  96.89%  97.24%  99.19%  99.69%  98.49%  97.6%  98.68% |
